# Effects of Visual and Auditory Feedback in Violin and Singing Voice Pitch Matching Tasks

**DOI:** 10.3389/fpsyg.2021.684693

**Published:** 2021-07-08

**Authors:** Angel David Blanco, Simone Tassani, Rafael Ramirez

**Affiliations:** ^1^Music and Machine Learning Lab, Department of Information and Communications Technologies, Universitat Pompeu Fabra, Barcelona, Spain; ^2^Multiscale and Computational Biomechanics and Mechanobiology Team, Department of Information and Communications Technologies, Universitat Pompeu Fabra, Barcelona, Spain; ^3^Music and Machine Learning Lab, Department of Information and Communications Technologies, Universitat Pompeu Fabra, Barcelona, Spain

**Keywords:** motor learning, feedback, violin, pitch-matching, auditory feedback, e-learning, music learning, singing

## Abstract

Auditory-guided vocal learning is a mechanism that operates both in humans and other animal species making us capable to imitate arbitrary sounds. Both auditory memories and auditory feedback interact to guide vocal learning. This may explain why it is easier for humans to imitate the pitch of a human voice than the pitch of a synthesized sound. In this study, we compared the effects of two different feedback modalities in learning pitch-matching abilities using a synthesized pure tone in 47 participants with no prior music experience. Participants were divided into three groups: a feedback group (*N* = 15) receiving real-time visual feedback of their pitch as well as knowledge of results; an equal-timbre group (*N* = 17) receiving additional auditory feedback of the target note with a similar timbre to the instrument being used (i.e., violin or human voice); and a control group (*N* = 15) practicing without any feedback or knowledge of results. An additional fourth group of violin experts performed the same task for comparative purposes (*N* = 15). All groups were posteriorly evaluated in a transfer phase. Both experimental groups (i.e., the feedback and equal-timbre groups) improved their intonation abilities with the synthesized sound after receiving feedback. Participants from the equal-timber group seemed as capable as the feedback group of producing the required pitch with the voice after listening to the human voice, but not with the violin (although they also showed improvement). In addition, only participants receiving real-time visual feedback learned and retained in the transfer phase the mapping between the synthesized pitch and its correspondence with the produced vocal or violin pitch. It is suggested that both the effect of an objective external reward, together with the experience of exploring the pitch space with their instrument in an explicit manner, helped participants to understand how to control their pitch production, strengthening their schemas, and favoring retention.

## 1. Introduction

The use of technology (e.g., mobile phones, computers, the internet) is widely used for a large number of different purposes related to music education (Zhukov, 2015). Some of the more demanded music learning apps are related to music theory, sight-reading, ear-training, and vocal training. Vocal training apps tend to offer real-time visual feedback of the performed pitch. However, despite the wide use of music education apps, such technologies are rarely employed in music schools where technology is usually restricted to audio/video recording and playback (Ramirez et al., 2015).

This research is part of TELMI (Technology Enhanced Learning of Musical Instrument Performance)^1^, a larger H2020 European project. In a previous study, we evaluated the effectiveness of augmented feedback in violin learning. In particular, we studied the effects of augmented feedback on pitch, motion-kinematics, and sound quality during the learning process of participants with no prior music experience (Blanco et al., 2021a). In the present study, we investigate the results of different types of feedback on intonation learning in singing and violin playing.

### 1.1. Background

#### 1.1.1. Real-Time Visual Feedback for Improving Intonation

Being able to play or sing in tune is an essential skill for most music students. That is probably the reason why the majority of the scientific literature about the effects of feedback in music learning has focused on intonation learning. Back to the beginning of the twentieth century, researchers from the University of Iowa developed a system to measure the pitch performed by participants and displayed it on a screen in real-time, allowing the participants to correct their performance instantaneously. They named their system Tonoscope (Seashore, 1902). Soon, a new generation of researchers started to study music performance and music learning using objective measures of sound such as frequency, intensity, and duration (for a review see Seashore, 1940). Some experiments attempted to show how training the ear with the visual feedback using the Tonoscope could result in a rapid improvement in pitch intonation and a transfer effect to new tones with different pitches (Seashore and Jenner, 1910; Knock, 1922; Brennan, 1926). Despite some methodological deficiencies (e.g., lack of control groups), these studies represent one of the first attempts to answer questions still relevant today regarding the use of feedback in music learning.

The Seashore's tonoscope was already available in the market in 1915. However, its use did not transcend outside the academic field. Some more recent approaches to characterize singing intonation skills were proposed by Welch with his schema theory of singing (Welch, 1985). From Welch's perspective, singing skills require external right/wrong feedback [also called knowledge of results (KR)] at the beginning of the learning process. The immediacy of this external feedback or concurrent KR is hypothesized to result in a more effective way of learning. Also, and in concordance with Schmidt's schema theory (Schmidt, 1975), the variability of practice may also be able to improve singing skills. Welch (1984) found how both real-time visual feedback with KR and variability of practice seemed to be an effective way to improve pitch-matching skills in children. These results were later replicated for melody production where, despite the fact that the participants worsened their accuracy at the time of receiving feedback, their results improved considerably in retention tests (Welch et al., 1989). Most importantly, real-time visual feedback without KR (that is, without right/wrong feedback) did not improve participants' performance in pitch-matching tasks (Welch, 1984). Similar results were recently found by Hutchins and Peretz (2012) in an experiment involving adult participants. This seems to evidence the importance of reward errors and objective measures for learning to sing.

Many singing apps have been proposed but few studies have attempted to evaluate their efficacy experimentally or in real learning contexts. For example, Wilson et al. (2005) evaluated with participants from different backgrounds and singing levels whether real-time visual feedback improved intonation in sung melodies more than discrete right/wrong feedback. They found that beginners benefit more from pitch real-time visual feedback than advanced singers, just as Welch hypothesized. They also found that participants' results tended to worsen at the moment of receiving feedback. Recently, Paney and Tharp (2019) evaluated the effects of real-time visual feedback with KR after 10 weeks of melody-singing training without finding significant differences with the control group. Some remarkable insights from that study come from the fact that participants using visual feedback tended to obtain better results than the control group although both groups improved. However, the removal of concurrent feedback led to a decay in performance for the experimental group whose retention scores were similar to those obtained by the control group. This drops the possibility that this type of feedback for improving singing skills could create dependency in the long term. On the other hand, both groups received KR at the end of each trial in the form of a score reflecting the overall accuracy of the trial. The lack of a control group without KR makes it hard to interpret if the improvement seen in participants could be related to KR or practice by itself.

In a recent study, Pardue and McPherson (2019) evaluated, both separately and combined, real-time auditory and visual feedback in violin intonation during four real-world violin lessons with beginners (adults and children). The real-time auditory feedback consisted of the pitch-corrected audio of a participant's playing to the nearest allowed pitch in the selected key (inspired by the tradition of students playing along with teachers). No statistical differences were found between each type of feedback, the combination of both nor the absence of feedback. However, their intonation was evaluated while using the technology and not in transfer or retention tests, also all the participants went through the different conditions instead of being separated into groups. Qualitative analysis and interviews of the participants seemed to point in the direction that the main problem of visual feedback was that it required visual attention. This could be the reason why some previous studies which evaluated the effects of real-time visual feedback in melodic production found a pattern of worsening results at the moment of receiving feedback (Welch, 1984; Wilson et al., 2005). On the other hand, some participants mentioned that the main problem with auditory feedback was that it did not provide information about in which direction errors should be corrected. Most participants seemed to prefer the combination of both types of feedback.

#### 1.1.2. Aural Feedback

Few studies have addressed the effectiveness of auditory feedback although their results remain contradictory. For example, Pfordresher and Brown (2007) found that hearing a synthesized voice concurrently with the singing of participants led to a detrimental effect on the absolute accuracy of poor-pitch singers but had a positive effect on good singers. On the other hand, Wise and Sloboda (2008) found that auditory feedback improved the performances of both “tone-deaf” and “non-tone-deaf” groups when singing familiar songs accompanied by the piano. Finally, Wang et al. (2012) found that the influence of accompanying auditory feedback (a synthesized piano) in song-singing tasks was negative. However, its effect was seen as positive for moderately poor-pitch singers in pitch-matching tasks.

One limitation of previous studies is that they did not study the possible effects of auditory feedback after removing it. Previous studies show that the effect of real-time visual feedback tends to worsen participants' performance in melody production despite improving it in retention conditions (Welch, 1984; Wilson et al., 2005). This effect could also occur with the use of concurrent auditory feedback. On the other hand, and more importantly, participants may be unable to use auditory feedback as KR. One of the reasons we would expect auditory feedback to improve singing skills is because it could be used by participants to recognize that they were not in tune. With the exception of Pardue and McPherson (2019), a common feature of almost all the previous studies was the use of synthesized sounds as auditory feedback. Hutchins and Peretz (2012) suggested that one of the main reasons for poor-pitch singing in their participants was due to a pitch-translation problem. The pitch-translation problem states that participants may not be able to “translate” the pitch from the timbre of the synthesizer to the timbre of their voice. This also leads us to reinterpret the studies that have used real-time visual feedback to improve singing skills: since many of them used synthesizers as reference tones to imitate, it could be argued that what they were really evaluating was the ability of the real-time visual feedback to help participants learn to translate the pitch of the synthesizer's timbre to the timbre of their voice.

Hutchins et al. recorded participants' voices singing different tones and asked them to do a self-matching task. They found improved results when matching their own voice (presumably due to timbral-similarity) but still worse results than when they used a knob-controller. Interestingly, experienced musicians who took part in the experiment were not able to distinguish voice tones differing by 30 cents (compared with the fact that they were able to distinguish synthesized tones with a difference of fewer than 10 cents). According to the authors, these results were due to a “vocal generosity effect.” The vocal generosity effect, which was addressed and confirmed in a posterior study (Hutchins et al., 2012), states that a higher degree of mistuning is necessary for listeners, both musicians, and non-musicians, to decide that sung tones were out-of-tune compared with the timbre of other instruments.

Recent work has addressed the effects of self-matching accuracy in melodies (Pfordresher and Mantell, 2014). In their first experiment, they found that participants were more accurate in imitating recorded melodies previously produced by themselves than recorded melodies produced by other participants. In their second experiment, they synthesized the pitch-time trajectories of the recorded melodies using a voice-like tone finding that the self-matching effect was independent of timbre. This self-advantage was also bigger for poor-pitch singers than accurate singers. According to the authors, poor-pitch singing is caused by a deficit of inverse modeling during vocal imitation where vocal-pitch patterns of participants are limited to the kinds of patterns they have produced in the past. However, the absolute error scores in the second experiment doubled those of the first experiment. Similar results were also found in previous research suggesting an important human-voice advantage in pitch imitation (Mantell and Pfordresher, 2013).

Humans, like some other animals (e.g., dolphins, whales, and birds), have the capacity to imitate arbitrary sounds through what has been commonly called auditory-guided vocal learning (Brown et al., 2004; Buccino et al., 2004; Fitch, 2006). Recent views, however, consider vocal learning to be separate phenomena from vocal imitation (Mercado III et al., 2014). Buccino et al. (2004) suggested that, when a motor action is coded in the mirror neuron system, it can be transferred to recombination of the viewed movements to replicate it. Thus, any action already presents in the mirror neuron system could be immediately replicated. Considering the significant exposure to human voices from the birth of any individual, human voices should be easier to replicate than other sounds and not only because of timbral similarity. Actually, Hutchins et al. found that participants from the self-matching task spent less time and required fewer trials than participants from the rest of the tasks. This could mean that participants managed to produce the required tone without hardly any effort. Also, the initial errors in the self-matching condition were much lower than in the slider condition. This implies that, in the slider condition, participants had to start from an almost arbitrary location of the pitch space letting auditory feedback guide their movement to the target note. That is, they did not develop a memory of the location where each pitch had to be found in the slider. However, in the self-matching condition participants seemed to be able to produce the required pitch without the need of starting from any arbitrary location. Both timbral cues and motor imagery may allow participants to recognize pitch due to an implicit/instrument-specific absolute pitch (Pfordresher and Halpern, 2013; Gelding et al., 2015; Reymore and Hansen, 2020).

### 1.2. Aims

In this study, we aim to evaluate in an experimental setup different modalities of feedback for learning to improve intonation in both the violin and the voice. Complete beginners with no musical experience took part in an experiment where they had to learn to maintain a stable sound with the violin while, additionally, were engaged in a pitch-matching task with their voices or the violin to study the effects of real-time pitch tracking and auditory feedback for this particular type of intonation exercise.

Inspired by the work of Hutchins and Peretz (2012), we designed a new experiment where, instead of using a slider, participants used a real instrument whose results will be compared to those of the voice. Beginners had to learn to translate the pitch from a synthesized pure tone used as a reference to a violin or their voice tone. Participants received help in the form of different types of feedback to improve their intonation skills which were posteriorly evaluated in a retention block. Beginners were randomly distributed into groups: the Control Group (CG) did not receive any type of help to improve their intonation abilities; the Feedback Group (FG) received real-time visual feedback with KR, and the Equal-Timbre Group (ETG) received similar timbre auditory feedback. By studying the retention effects of both the FG and ETG groups we expected to isolate the effects of “external reward” in learning pitch-matching abilities while comparing the effects of auditory feedback in the form of timbre-similarity in different instruments. Finally, we also created an Expert Group (EG) formed by expert violinists.

In general terms, in this study we seek to answer the following questions:

1. Does real-time visual feedback improve participant's pitch-matching abilities with a synthesized tone for both violin and singing voice?
2. How does timbre-similarity affect pitch-matching abilities in violin and singing voice?
3. Does timbre-similarity help participants learn how to translate the pitch from a synthesized sound to that of their voice or instrument?
4. How do real-time visual feedback and timbre-similarity affect participants' retention scores?

We expected that real-time visual feedback would positively impact the results of the FG as has already been shown in previous research. However, previous research tended to measure improvements only in pitch accuracy (that is, the error in cents from the desired notes). As in Hutchins and Peretz (2012), we decided to also collect the number of correct notes by considering a note correct if it is within 50 cents of the target pitch.

If timbre-similarity and imitation skills influence the results of pitch-matching tasks, we would expect it would be easier for participants from the ETG to imitate human voice pitches than violin pitches. We would also expect that ETG participants would find voice pitches faster than FG participants.

We followed some of the methodological procedures proposed during the Seattle International Singing Research Symposium (Demorest et al., 2015). All the data used in the current study (raw data, wav files, and statistics) are publicly available in Zenodo (Blanco et al., 2020, 2021b).

## 2. Methods

### 2.1. Participants

Fifty-seven participants with no prior violin playing experience and no musical experience with other instruments (34 female and 23 male) were recruited from the Pompeu Fabra University campus to participate in the study. In addition, 15 expert violinists [EG; 8 women, 7 men; mean age: 32.4 (10.06); mean years experience: 18.6 (5.53)] were recruited from both the university campus and different music schools and conservatories in Barcelona. Participants conceded their written consent and procedures were approved by the Conservatoires UK Research Ethics committee on 04/04/2017, following the guidelines of the British Psychological Society.

Participants filled a questionnaire about their musical skills, main instrument, and years of music training. They also performed a pitch discrimination task (PDT) before (pre) and after (post) the experiment (Musicianbrain, 2021). Those participants who got pitch discrimination thresholds above 18 Hz in both pre and post-tests were asked to realize the Brams Online Test for musical abilities (Peretz et al., 2008). Those participants who got scores below 70% in both the first and third sections of the Brams test were labeled as “possible amusics” and removed from the experiment. We discarded one participant who reported after the experiment being unable to take pleasure in music. She also failed the first block from the Amusia test. Finally, we also discarded all the participants who reported having played a musical instrument for more than 1 year.

Beginner participants were randomly divided into three different experimental groups: the Feedback Group [FG; 9 female, 6 male; mean age: 27.93 (4.33)], the Control Group [CG; 11 female, 4 male; mean age: 27.83 (4.95)], and the Equal Timbre Group [ETG; 10 female, 7 male; mean age: 30.76(8.3)]. The study was carried out in the recording studio located in the Information and Communication Technologies Engineering (ETIC) department of the Universitat Pompeu Fabra, Barcelona.

### 2.2. Materials

#### 2.2.1. Learning Materials

Before the experiment, basic information about violin playing techniques like stance, violin position, bow position, and grip was delivered to the beginner participants through a 6-min didactic Youtube video of a professional violinist^2^. The video covered violin technique aspects such as bow-string *contact point*, bow speed-force relationship, and bow angle. The video included an example of how to perform full bow exercises (alternation of up and down movements using the full length of the bow) focusing attention on bowing parallel to the bridge and how to move the wrist of the right hand to achieve a straight bow movement. The experts visualized only the last part of the explanation to make sure they understood the task.

#### 2.2.2. Providing Visual Feedback With SkyNote

**Figure 1:**
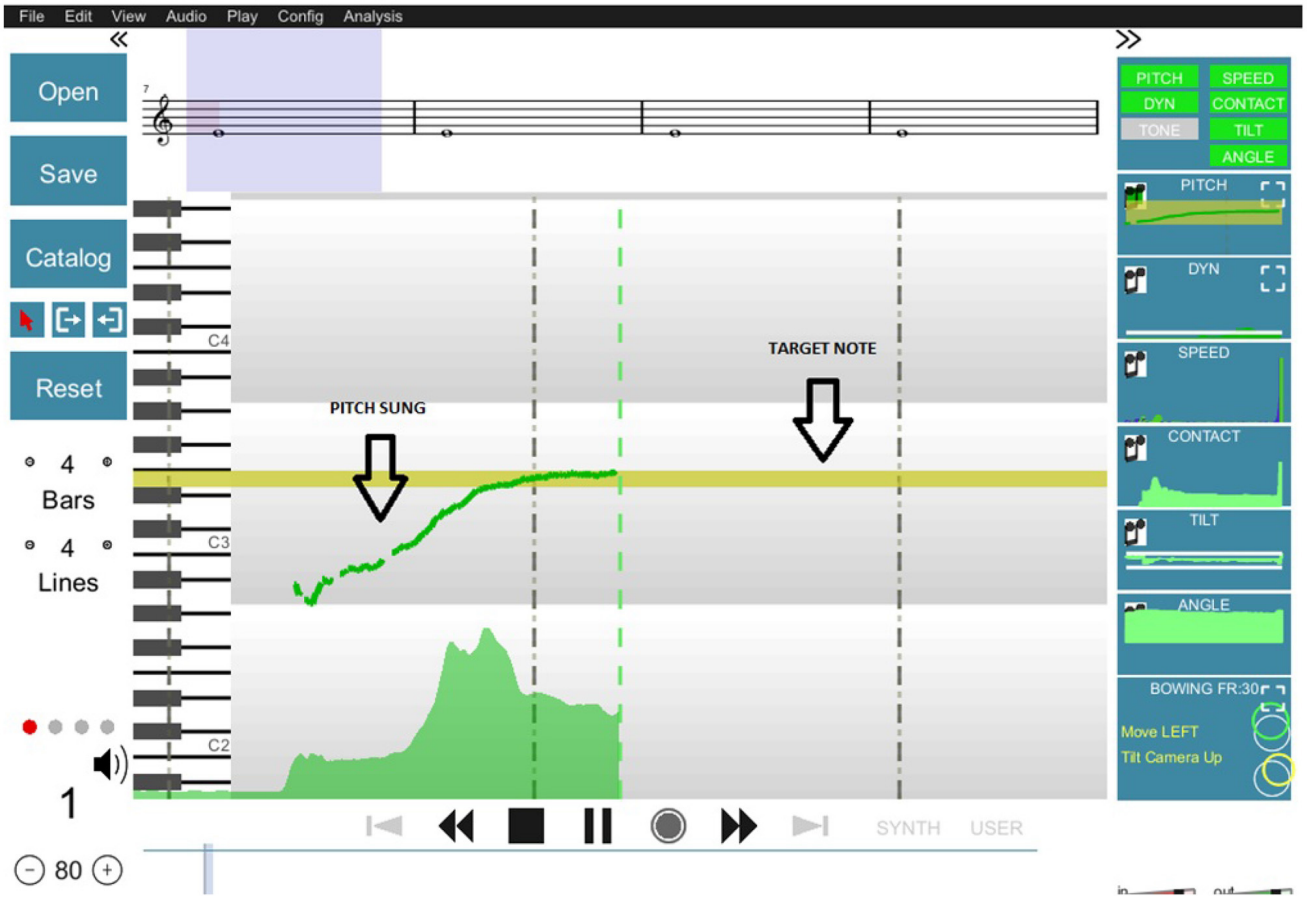
Visual display of the tool used to offer real-time feedback of pitch production. The target note, which in this case is an E3, is represented on the screen as a yellow bar. The produced pitch by the participant is drawn in green at the center of the screen at the moment of the production and displaced to the left of the screen over time.

SkyNote, the system we used to deliver real-time feedback to participants, is one of the main outcomes of the TELMI Project. SkyNote is an integrated system that combines different technologies for real-time feedback on pitch, intonation, dynamics (Mayor et al., 2009), kinematics (Vamvakousis et al., 2018), and tone quality (Giraldo et al., 2019). This feedback can be displayed in customized widgets or directly on the musical score, allowing for real-time experimentation and overall performance evaluation. For this experiment, we presented feedback of a single performance aspect at a time. In Figure 1, you can see the display used for the real-time feedback on pitch, intonation, and dynamics. The target note appears in the yellow bar on the screen while the performed note is represented by a green line (for more details see Mayor et al., 2009). Five different musical notes were reproduced triggering a reference synthetic sound which consisted of a pure tone at the following frequencies: D#4(311.13 Hz), E4(329.63 Hz), F4(349.23 Hz), F#4(369.99 Hz), G4(392.00 Hz) for most female participants and an octave below for most male participants. Different octaves were chosen if needed to fit the vocal range of participants independently of their gender.

We used a condenser microphone (Behringer-C3, 2013) to record the audio during the session, a NUC computer to run SkyNote, and two screens, one to deliver feedback to the participant and the other one for the experimenter.

### 2.3. Experimental Procedure

Before starting the experiment all groups of participants took part in a practice session monitored by the experimenter. In that practice session, they were instructed on violin technique, bow position, stance, and bow grip through a Youtube video which explained some of the most important concepts required to perform the full bow exercises correctly together with audio-visual examples. A full bow exercise consisted of the alternation of two up and down bowing movements using the full length of the bow with the goal of producing a stable and clear sound. Participants could play while watching the video and explore creating sound with the violin. They could also rewatch different parts of the video while practicing. Participants were informed orally by the experimenter about the violin technique aspects to take into account. These variables were also explained in the Youtube video: *bow skewness* (bowing parallel to the bridge), *contact point* (measured as bow-bridge distance), *inclination* (taking care of not playing the other strings during the movement with the bow), *pitch stability* (related to avoiding scratchy sounds), and *dynamic stability* (trying to maintain the energy of the sound stable during the whole exercise, even during up-to-down or down-to-up changes). More details about how these variables were computed can be found in Blanco et al. (2021a). They were also encouraged to explore how the produced pitch changes when they move their finger down the fingerboard. The experimenter verified that all participants were able to perform this task correctly before continuing with the experiment. The duration of this practice session was around 16 min (6 min video + 10 min practice). In order, to find the vocal range for each participant, they were asked to perform some singing warm-up exercises such as sustaining a single comfortable pitch for several seconds. Participants were also asked to make a sweep from the lowest note they could produce to the highest one and another sweep from their highest note to their lower note to ensure that their range covered the space of all the target notes.

The experiment consisted of three blocks: *Baseline, Acquisition*, and *Transfer*. The *Baseline* was equal for all groups of participants and consisted of a pitch-matching exercise with five different notes alternating between the *violin* and the *voice* condition. At the beginning of the *Baseline* block, one of five target notes was produced with the reference synthetic sound for five seconds (synth-matching task) while participants were not allowed to play or sing. In the *violin* condition, participants had to locate in the fingerboard of the D string the target note by displacing their index finger across the fingerboard while producing sound with the bow. Participants could start from any location on the fingerboard. Once the note was located, they were asked to perform a full bow exercise on that specific location. The note was then reproduced again for five seconds giving participants the possibility of changing their decision. Whether they decided to change or not, they had to perform another full bow exercise. In the *voice* condition participants repeated the same procedure described above but using their voice. The *voice* and *violin* conditions were alternated in random order for each note. That is, sometimes starting with the voice and sometimes starting with the violin.

After the *Baseline* block and before the *Acquisition* block, participants rewatched the instructional video and remained about the main variables that will be used to evaluate their performance. They were also instructed about the procedure of the *Acquisition* block. Real-time visual feedback was presented and explained to the FG. Participants rested around 5 min in between blocks.

**Figure 2:**
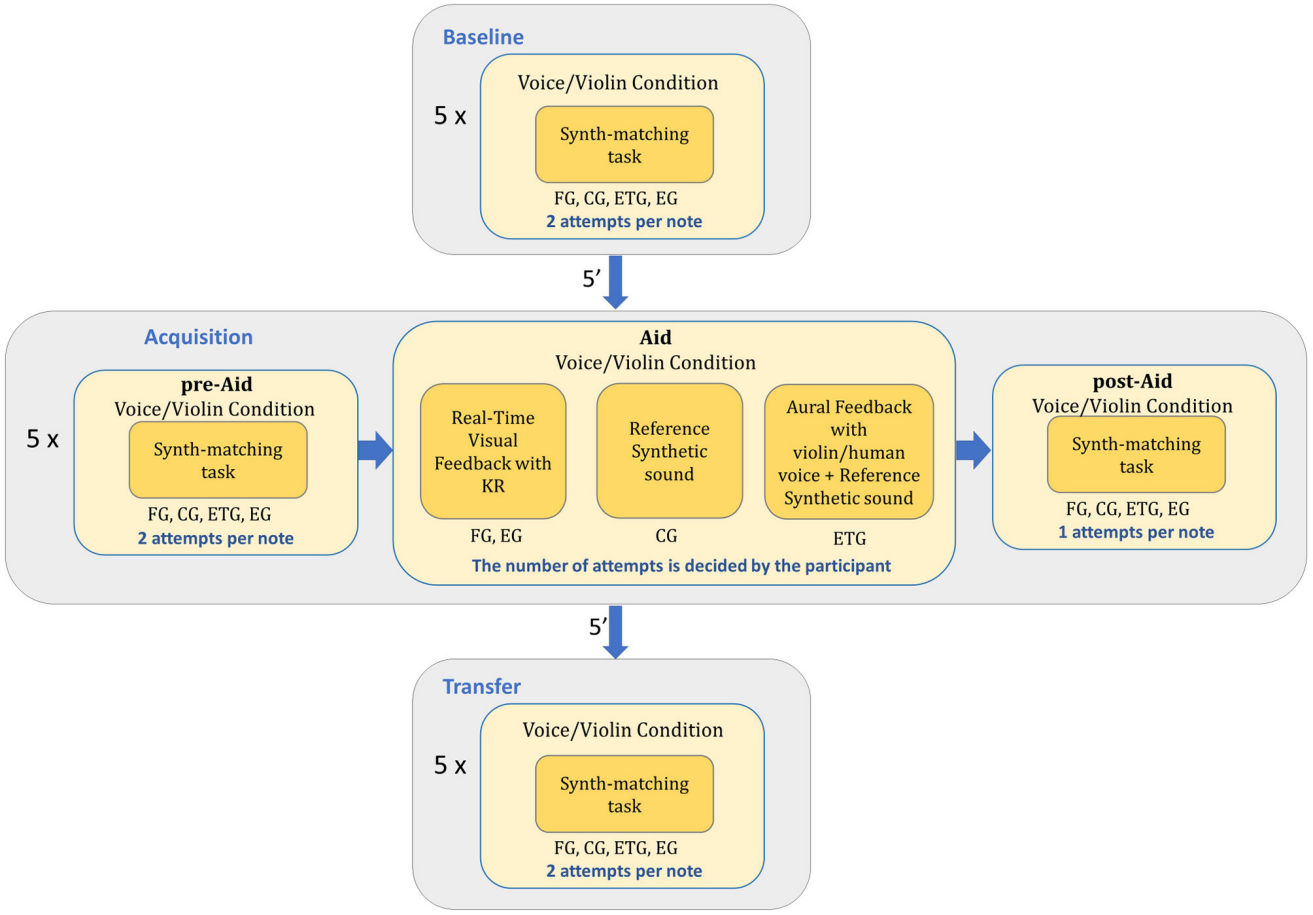
Diagram with the different blocks of the experiment and the different conditions each group of participants went through.

As in the *Baseline* block, the *Acquisition* block also consisted in a synth-matching exercise with the same five notes presented in the *Baseline* and an alternation between the violin and the voice. First, participants tried to match the corresponding pitch in a synth-matching task with two attempts just like in the *Baseline*. This was called the *Acquisition pre-Aid* condition. After the second attempt, the *Acquisition Aid* condition started. The *Acquisition Aid* condition differed between the three different groups of beginners. Visual feedback was provided to the FG on how far their performed note was from the target note. Using the feedback participants in the FG could modify their performed notes and hear the reference synthetic sound as many times as they needed. After that, feedback was removed and participants performed a synth-matching task for the same note (*Acquisition post-Aid*). On the other hand, in their *Acquisition Aid* condition, the ETG was able to modify the performed note with matched-timbre auditory feedback of the corresponding target note. Participants were allowed to request both the recordings and the reference synthetic sounds as many times as they needed (either because they were satisfied or decided to give up). CG participants only had the option to hear the reference synthetic sound and change their performed note, as many times as needed. Following this, both the ETG and CG repeated a last synth-matching task for the same note (*Acquisition post-Aid*). Finally, the experts received real-time visual feedback in the *Acquisition Aid* condition like the FG and performed a last synth-matching task. The reason for that is because we wanted them to fill a questionnaire with their opinion about SkyNote at the end of the experiment (see Blanco et al., 2021a for the answers to the questionnaires). As in the rest of the conditions, participants were not allowed to play or sing during any type of sound reproduction in the *Acquisition-Aid* condition. Summarizing, we can divide the *Acquisition* block into three different conditions: the *Acquisition pre-Aid*, the *Acquisition Aid*, and the *Acquisition post-Aid* (see Figure 2).

After the *Acquisition* block participants rested for 5 min. Then, the *Transfer* condition started. The *Transfer* condition was the same as the *Baseline* condition but with a different order of notes and alternations between the violin and the voice condition (see Figure 2). The order of the notes and alternations between the violin and voice condition was randomized as in the other conditions.

After the *Acquisition post-Aid* condition with the violin half of the participants chosen randomly performed one full bow exercise while receiving real-time feedback about sound quality and one more full bow exercise while receiving real-time feedback on bow kinematics. The other half were also asked to perform the two full bow exercises in a row. In the first one, they were explicitly asked to pay attention to the sound quality feedback and in the second one to the kinematic feedback. These results are presented in Blanco et al. (2021a).

### 2.4. Intonation Analysis

The Tony software (Mauch et al., 2015) was used to extract information from pitch accuracy from the raw audio of violin and voice exercises. However, it was necessary to visually inspect all the events to ensure the correct operation of the pitch detection algorithm. This data was posteriorly processed in Matlab (MATLAB, 2010) and analyzed in Spss (IBM, 2011).

#### 2.4.1. Pitch Detection

The audios for each condition were recorded in a .wav file by the software at a sample rate of 44,100 Hz. The Tony software was used to extract the pitch of all performed (violin and singing voice) notes (Mauch et al., 2015). Tony is based on the pYIN method for automatic pitch estimation and note tracking (Mauch and Dixon, 2014) together with custom methods for interactive reestimation. It outputs discrete notes on a continuous pitch scale based on the Viterbi-decoding of an independent Hidden Markov Model. This method is particularly robust to small and short pitch variations. If one variation is big and long enough, like in the possible case of one participant accidentally hitting another string, the Tony software considers that a pitch transition occurred, and returns two different pitch estimations separated in time. A visual inspection of all the events was, thus, also necessary to ensure correct pitch extraction.

The pitch performed by each participant was converted to cents using the target note as a reference. To avoid octave errors, those sung or played pitches with a value >+600 or lower than −600 cents were recomputed to a different octave. Finally, we computed the absolute value of the errors. We also considered the number of correct notes, that is, those pitches sung with an error of <50 cents (half semitone).

#### 2.4.2. Violin Technique Analysis

Before starting with the intonation analysis we evaluated whether violin technique could have exerted an influence on the pitch-matching skills of our beginner participants. For that purpose, we looked for possible correlations between beginners' average absolute pitch errors and their technique (both in terms of sound quality and gestures). For sound quality we computed two descriptors which have been proven to be useful in previous research: *dynamic stability* and *pitch stability* (Romaní et al., 2015; Blanco and Ramirez, 2019; Giraldo et al., 2019; Blanco et al., 2021a). We also evaluated the participants' gestural technique using one kinematic descriptor: *bow skewness*. This descriptor represents the angle of the bow with respect to the violin bridge (the closer to zero the better) which is considered to be a common prerequisite to achieve a good sound. More information about how those descriptors were computed can be found in Blanco et al. (2021a). We also evaluated for possible differences between groups in their performance across blocks with one mixed-design 3 × 4 with Group (CG, ETG, FG) as between-subject factor, and Condition (*Baseline, Acquisition pre-Aid, Acquisition post-Aid*, and *Transfer*) as within-subject factor. Finally, to ensure that the amount of improvement was not significantly bigger for one group than for the others, we performed three independent t-tests of the relative difference between the Transfer and the Baseline for each one of the descriptors applying Bonferroni correction.

#### 2.4.3. Behavioral Analysis at the Baseline

We evaluated the behavior of beginner participants in the *Baseline* condition and compared it with the experts. To ensure that participants were trying to match the target pitches we compared their average error in cents over the five notes. Both for the violin and the voice. We also verified if there was a correlation between the frequency of the target notes and the frequency of the produced notes. This helped us to evaluate whether a target pitch was higher than the previous one, the direction of the produced pitch was also higher compared with the previous one.

We also evaluated the possibility that some notes could be more difficult to match than others. We performed two 2 × 2 repeated measures analyses with Instrument (violin, voice) and Note as within-subject factors for beginners and experts. Posteriorly, we evaluated if participants tended to correct their errors in the correct direction in their second attempt. We performed two more 2 × 2 repeated measures analysis with Instrument (violin, voice) and Attempts (first and second attempt).

Finally, we also studied if there was any significant trend to flat or sharp notes in the direction of the errors of both beginners and experts when playing violin or singing. For that purpose, we performed four one-sample *t*-tests.

#### 2.4.4. Analysis of the Effects of Feedback

Finally, we performed four more different analyses of the data. One for the error in cents, another one for the number of correct notes, another one for the time in seconds they spent in *Acquisition post-Aid* and finally, another one for the number of times participants from the ETG and CG requested auditory feedback in *Acquisition Aid*.

To study the impact of the different types of feedback in each modality we performed for each analysis one 4x (4 × 2) mixed-design with Group (CG, ETG, FG, and the experts) as between-subject factor, and Condition (*Baseline, Acquisition pre-Aid, Acquisition post-Aid*, and *Transfer*) and Instrument (violin and voice) as within-subject factors. *Post-hoc* tests using the Tukey method for multiple comparisons were performed between the groups of participants to compare their results. We also performed a 4 × 2 mixed-design with Group as between-subject factor and Instrument as the within-subject factor for the analysis of duration and a 2 × 2 mixed-design with Group (ETG and CG) and Instrument for the number of feedback requests.

For those analyses that showed a significant interaction between Condition and Group, a posterior simple main effect analysis was performed on each group to find out which conditions were causing the interaction. Pairwise comparisons tests were performed between the conditions using the Bonferroni correction.

Finally, we removed two participants from the FG and two more from the expert group because they were labeled as outliers. After removing the outliers all the data passed the assumptions of normality required to perform the tests. All the results presented in the following sections were Greenhouse-Geisser corrected.

## 3. Results

### 3.1. Sound Quality and Bow Technique With the Violin

All groups of participants experienced improvements in violin technique in all the measured descriptors through the different blocks of the experiment. The mixed-analysis showed a significant effect of Condition for *pitch stability, F*_(1.97)_ = 16.27, *p* < 0.0001, η^2^ = 0.34, for *dynamic stability, F*_(2.79)_ = 5.17, *p* = 0.003, η^2^ = 0.14, and for *bow skewness F*_(2.21)_ = 5.10, *p* = 0.007, η^2^ = 0.14. No significant Condition*Group interaction was found. No significant differences were found in the relative amount of improvement at the end of the session between groups.

Finally, we did not found significant correlations between the absolute error in cents beginners made with the violin in each condition with the value of each one of the three descriptors used to measure violin technique (*pitch stability, dynamic stability, bow skewness*).

### 3.2. Behavioral Results at the Baseline

**Figure 3:**
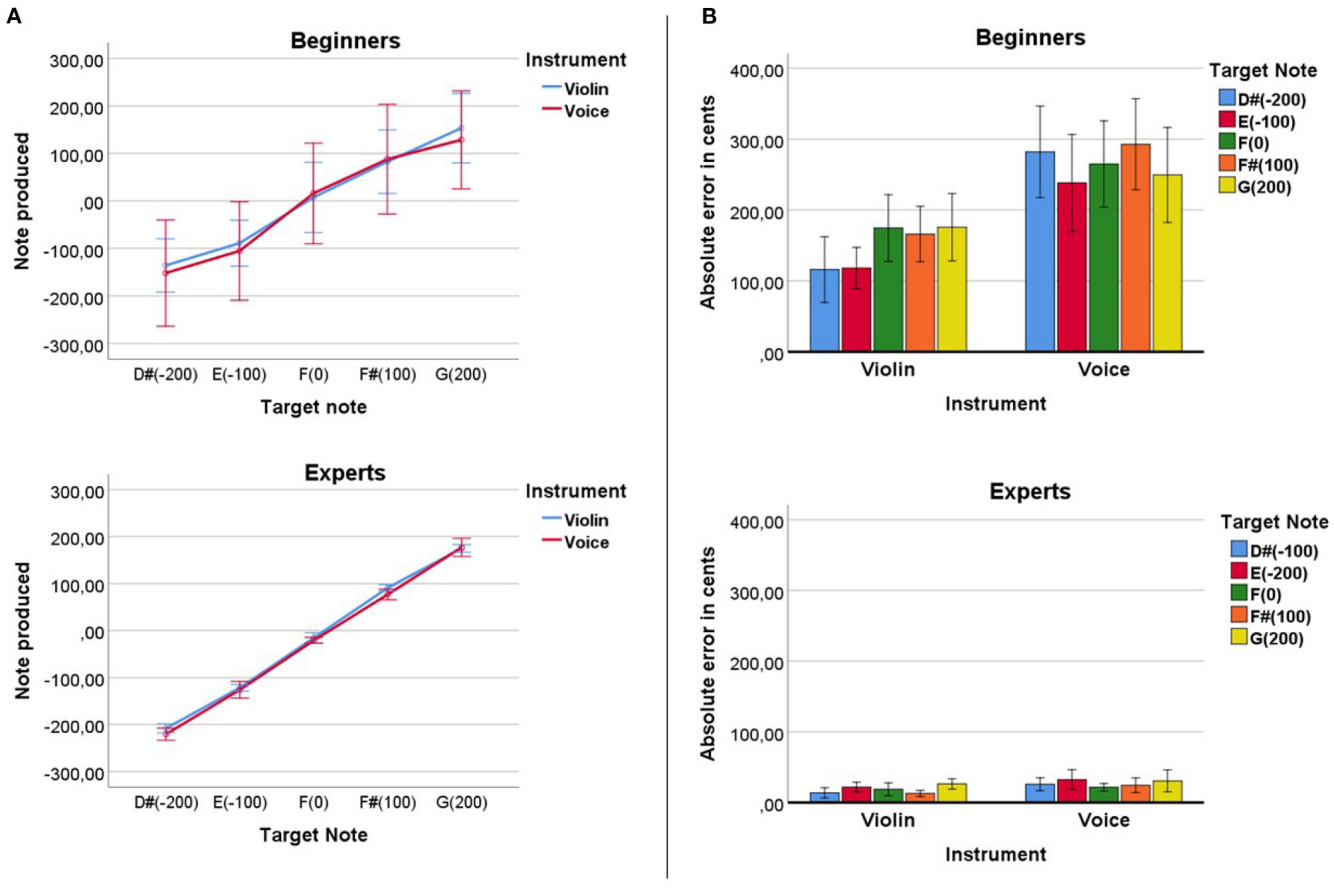
**(A)** Comparison between the target and produced note of beginners and experts both with the violin and with the voice. **(B)** Absolute error in cents for each target note. Errors produced with the voice were significantly bigger than errors produced with the violin both for beginners and experts. No significant differences were found between the error produced at each note.

We found significant correlations at the *Baseline* condition between the tone that beginners produced and the target tones of the experiment when using the voice, *R*^2^ = 0.318, *p* < 0.0001 and the violin, *R*^2^ = 0.47, *p* < 0.0001 (see Figure 3A). As expected, the experts showed stronger correlations between their produced tone and the target tones of the experiment for the voice, *R*^2^ = 0.98, *p* < 0.0001 and for the violin, *R*^2^ = 0.99, *p* < 0.0001.

Beginners showed an absolute average error of 150 cents (SD = 12.56) with the violin. Errors for the voice tended to be bigger than for the violin (see Figure 3B). On average beginners showed an error with the voice of 271 cents (SD = 23.83). The mixed analysis for beginners showed a significant effect of Instrument (voice > violin), *F*_(1)_ = 20.37, *p* < 0.0001, η^2^ = 0.35. No significant effects of Note nor an Instrument*Note interaction were found. Experts showed an absolute average error of 18 cents (SD = 1.59) with the violin and an absolute average error of 26.82 cents (SD = 2.98) for the voice. The mixed analysis for experts showed a significant effect of Instrument (voice > violin), *F*_(1)_ = 5.93, *p* < 0.032, η^2^ = 0.33. No significant effects of Note nor an Instrument*Note interaction were found.

Beginners tended to improve their accuracy in the second attempt when compared with the accuracy of their first attempt by 11.88 cents (SD = 5.63). The repeated measures analysis showed a significant effect of Instrument (voice > violin), *F*_(1)_ = 18.73, *p* < 0.0001, η^2^ = 0.35, and of Attempts (second < first), *F*_(1.00)_ = 4.43, *p* < 0.041, η^2^ = 0.09. No significant Instrument*Attempts interaction was found. Experts did not show any significant differences between Attempts neither at Instrument.

Finally, beginners did not show any tendency toward sharp or flat errors neither in the violin nor in the voice as revealed by the one-sample *t*-tests. On the other hand, experts did show a flat trend both with the violin, *t*_(12)_ = −7.5, *p* < 0.0001, and with the voice, *t*_(12)_ = −6.75, *p* < 0.0001. On average, the error in cents of the experts while using the violin was −16 cents (SD = 7.69) and −22 cents (SD = 12.15) when using the voice.

### 3.3. Pitch Matching Across Blocks

**Figure 4:**
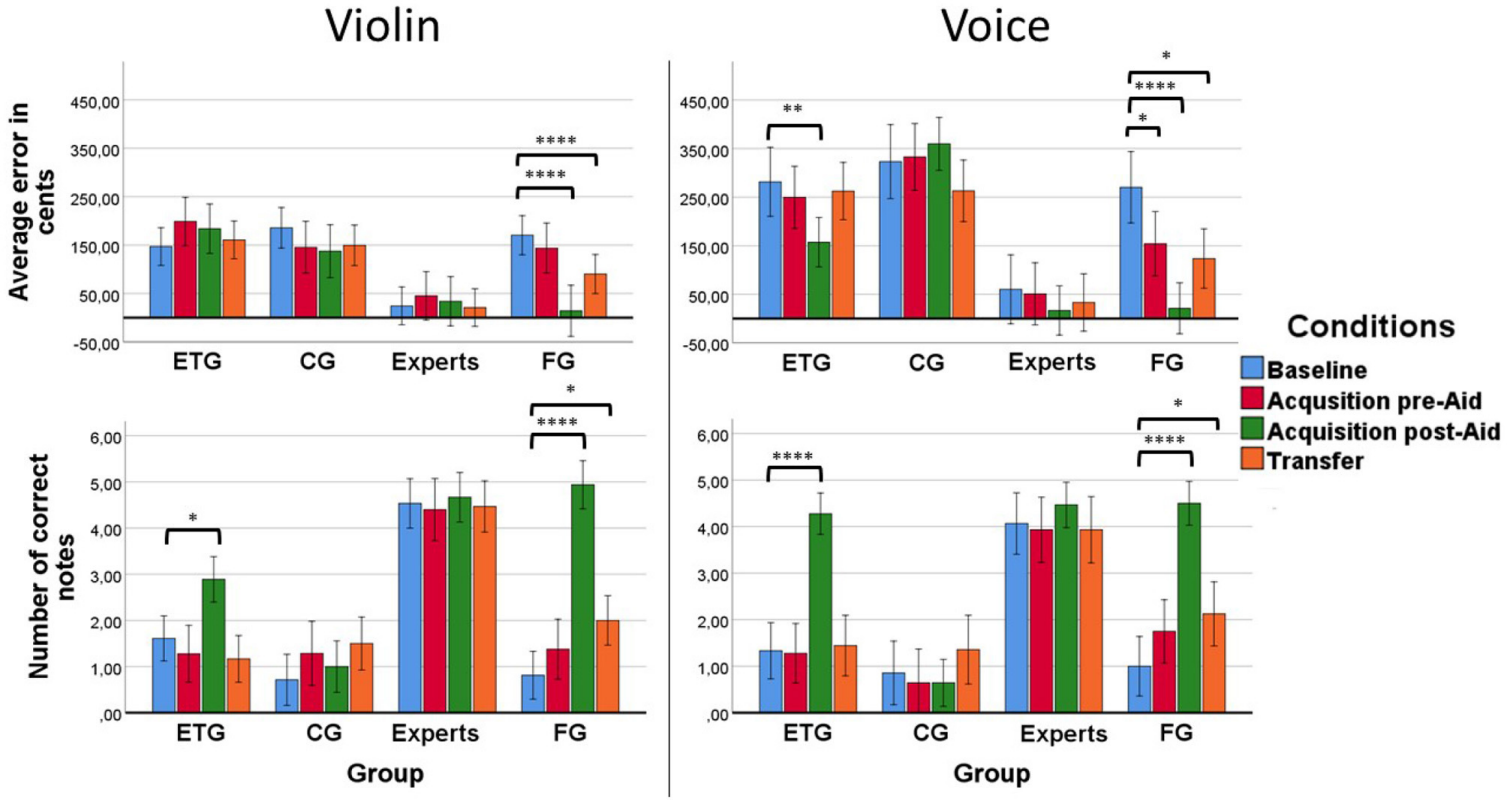
**(Upper left)** Results of pitch-matching accuracy in the violin condition for each group of participants. Only the FG improved significantly their results compared with the *Baseline* at both the *Acquisition post-Aid* and *Transfer*. **(Upper right)** Results of pitch-matching accuracy in the voice condition for each group of participants. The FG improved significantly their results at the *Acquisition pre-Aid, Acquisition post-Aid*, and *Transfer* conditions. The ETG improved significantly their results at the *Acquisition post-Aid*. However, that improvement was not retained to the rest of the following conditions. **(Inferior left)** Results of the number of correct notes in the violin condition for each group of participants. The FG improved significantly their results compared with the *Baseline* at both the *Acquisition post-Aid* and *Transfer*. The ETG improved significantly their results only at the *Acquisition post-Aid*. **(Inferior right)** Results of the number of correct notes in the voice condition for each group of participants. As in the violin condition, the FG improved significantly their results compared with the *Baseline* at both the *Acquisition post-Aid* and *Transfer* while the ETG improved significantly their results only at the *Acquisition post-Aid*. (*p < = 0.05, **p < = 0.01, ****p < = 0.0001).

Results showed that all groups of participants showed larger errors for the voice than for the violin. As expected, the error in cents of the experts was on average much lower (M = 18.39, SD = 18.18 cents with the violin and M = 34.46, SD = 29.16 cents with the voice) than the rest of the groups of beginners (M = 135, SD = 17 cents with the violin and M = 217.33, SD = 27.32 cents with the voice). See Figure 4 for a summary of the results. *Post-hoc* tests showed significant differences between the experts with the rest of the groups (experts < CG, *p* < 0.0001; experts < ETG, *p* < 0.0001; experts < FG, *p* = 0.005). Also, results from the FG differed significantly from both the ETG and the CG (FG < ETG, *p* = 0.006; FG < CG, *p* < 0.0001).

The FG improved their results through the different conditions at both the violin and the voice (see Figure 4). The ETG showed different behavior for the voice than for the violin at the *Acquisition post-Aid*. We found that, on average, the error with the voice decreased 120.80 cents (SD = 131.56) compared to the *Baseline* when the ETG received aid in the form of a human voice. That decrease was not seen in the violin condition.

The Univariate tests of within-subject effects for error in cents showed a significant effect of Instrument (voice > violin), *F*_(1)_ = 16.20, *p* < 0.0001, η^2^ = 0.23, and an Instrument*Group interaction, *F*_(3)_ = 2.93, *p* = 0.041, η^2^ = 0.14. Also significant effects of Condition, *F*_(2.51)_ = 20.6, *p* < 0.0001, η^2^ = 0.28, and a Condition*Group interaction, *F*_(7.54)_ = 7.59, *p* < 0.0001, η^2^ = 0.29. We also found an Instrument*Condition*Group, *F*_(6.34)_ = 3.03, *p* < 0.008, η^2^ = 0.144. We did not found an Instrument*Condition interaction.

The repeated measures for each group revealed a significant effect of Condition in the univariate tests of within-subject effects for the ETG, *F*_(2.72)_ = 4.17, *p* = 0.019, η^2^ = 0.20, and a Condition*Instrument interaction, *F*_(2.53)_ = 6.69, *p* = 0.001, η^2^ = 0.28. Pairwise comparisons tests showed significant results for the ETG between the *Baseline* and the *Acquisition post-Aid* conditions for voice (*Baseline* > *Acquisition post-Aid, p* = 0.008). The CG showed only a significant effect of Instrument (voice > violin), *F*_(1)_ = 12.03, *p* = 0.004, η^2^ = 0.48. Finally, the FG showed a significant effect of Condition, *F*_(1.9)_ = 18.73, *p* < 0.0001, η^2^ = 0.748. Pairwise comparisons for the violin showed significant results for the FG between both the *Baseline* and the *Acquisition post-Aid* and *Transfer* conditions (*Baseline* > *Acquisition post-Aid*; *Baseline* > *Transfer, p* < 0.0001 for both tests). Pairwise comparisons for the voice showed significant results for the FG between the *Baseline* and the rest of the conditions: the *Acquisition pre-Aid, Acquisition post-Aid*, and *Transfer* (*Baseline* > *Acquisition pre-Aid, p* = 0.048; *Baseline* > *Acquisition post-Aid, p* < 0.0001; *Baseline* < *Transfer, p* = 0.045).

The FG produced better results than the ETG at the *Acquisition post-Aid* condition. The FG showed an average error of 14.73 cents (SD = 2) for the violin and an average error of 20.45 cents (SD = 2.27) for the voice. On the other hand, the ETG showed an average error of 169.24 cents (SD = 31) for the violin and an average error of 145.36 cents (SD = 32.33) for the voice. Independent samples *t*-test showed significant effects at the *Acquisition post-Aid* condition between the FG and ETG for the violin (FG < ETG), *t*_(29)_ = 4.17, *p* < 0.0001, and for the voice (FG < ETG), *t*_(29)_ = 3.26, *p* = 0.003. No significant effects were found in a paired samples t-test between the voice and violin condition for the ETG.

The FG also improved their results more than the ETG at the *Transfer* condition in relation to the *Baseline* (*Transfer* - *Baseline*). The FG showed an average improvement of 81.17 cents (SD = 51.9) for the violin and an average improvement of 105.31 cents (SD = 168.66) for the voice. On the other hand, the ETG showed an average improvement of 24 cents (SD = 123.4) for the violin and no improvement for the voice (M = −11, SD = 15). Independent samples *t*-test showed significant differences at the degree of improvement between the FG and ETG for the violin (FG < ETG), *t*_(29)_ = −3.29, *p* < 0.003, and for the voice (FG < ETG), *t*_(29)_ = −2.41, *p* = 0.022.

### 3.4. Correct Notes Across Blocks

Despite the main differences found in accuracy between the voice and the violin across blocks, we did not find big differences regarding the number of correct notes. On average, beginners made in the *Baseline* an average number of 1.02 correct notes (SD = 1.23) with the voice and an average number of 1.08 correct notes (SD = 1.04) with the violin. On the other hand, experts made an average number of 4.06 correct notes (SD = 1.48) with the voice and an average number of 4.53 correct notes (SD = 1.3) with the violin. We did not find significant effects of Instrument or Instrument*Group or Instrument*Condition interaction.

The main difference compared with accuracy results across was seen in participants of the ETG. We found an improvement in the number of correct notes at the *Acquisition post-Aid* not only for the voice (M = 4.28, SD = 0.24) but also for the violin (M = 2.83, SD = 0.33). The improvement for singing voice, however, was still bigger than the improvement for violin and resembled those of the experts (M = 4.46, SD = 0.18) or the FG (M = 4.38, SD = 0.26) at the moment of receiving real-time visual feedback. Univariate tests of within-subjects effects for correct notes showed a significant effect of Condition, *F*_(2.38)_ = 56.51, *p* < 0.0001, η^2^ = 0.51, a Condition*Group interaction, *F*_(7.15)_ = 18.24, *p* < 0.0001, η^2^ = 0.50, and an Instrument*Condition*Group interaction, *F*_(8.49)_ = 3.30, *p* = 0.001, η^2^ = 0.155.

The repeated measures for each group revealed a significant effect of Condition in the univariate test of within-subject effects for the ETG, *F*_(1.85)_ = 54.58, *p* < 0.0001, η^2^ = 0.76, and interaction between Condition*Instrument, *F*_(2.32)_ = 7.46, *p* = 0.001, η^2^ = 0.355. Pairwise comparisons tests showed significant results between the *Baseline* and the *Acquisition post-Aid* conditions for singing voice (*Baseline* < *Acquisition post-Aid, p* < 0.0001) and for violin (*Baseline* < *Acquisition post-Aid, p* = 0.038). The FG showed a significant effect of Condition, *F*_(2.11)_ = 63.28, *p* < 0.0001, η^2^ = 0.841. Pairwise comparisons for the violin showed significant results between both the *Baseline* and the *Acquisition post-Aid* and *Transfer* conditions (*Baseline* < *Acquisition post-Aid, p* < 0.0001; *Baseline* < *Transfer, p* = 0.036). We also found a significant effect of Instrument for the experts (violin < voice), *F*_(1)_ = 6.09, *p* = 0.03, η^2^ = 0.337.

The FG improved their results more than the ETG at the *Acquisition post-Aid* condition for the violin condition (difference: M = 2.09, SD = 0.39) although their results in singing voice were similar (difference: M = 0.1, SD = 0.36). Independent samples *t*-test showed significant effects between the FG and ETG for the violin at the *Acquisition post-Aid* (FG > ETG), *t*_(29)_ = −5.34, *p* < 0.0001. No significant differences were found for the voice. The ETG improved more in the *Acquisition post-Aid* with the voice than with the violin (difference: M = 1.4, SD = 1.75). A paired samples t-test showed significant results for the ETG between singing voice and violin in the *Acquisition post-Aid* (voice > violin), *t*_(17)_ = −3.48, *p* = 0.003).

The FG improved their results more than the ETG at the *Transfer* condition in relation to the *Baseline* (*Transfer* - *Baseline*). The FG showed an average improvement of 1.38 correct notes (SD = 1.5) for the violin and an average improvement of 0.92 correct notes (SD = 1.03) for the voice. On the other hand, the ETG showed no improvement for the violin (M = −0.38, SD = 1.57) and an average improvement of 0.16 correct notes (SD = 1.09) for the voice. Independent samples *t*-test only showed significant differences at the degree of improvement between the FG and ETG for the violin (FG > ETG), *t*_(29)_ = −2.31, *p* = 0.028.

### 3.5. Duration of Acquisition Aid

**Figure 5:**
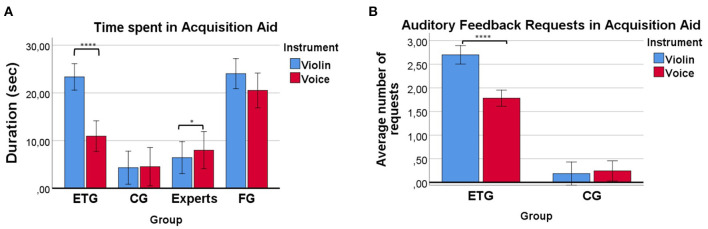
**(A)** Duration of the *Acquisition Aid* for each group. Participants from the FG and ETG spent more time than the control group trying to match the required pitch probably because of the aid they were receiving. The ETG spent significantly less time trying to match pitches with the voice than with the violin. **(B)** Number of times participants requested auditory feedback. Participants from the ETG tended to request more times the auditory feedback at the violin condition than at the voice condition. On the other hand, participants from the CG tended to not request more feedback from the synthesizer. *p <= 0.05, ****p <= 0.0001.

Results for duration showed how participants from the ETG tended, on average, to spend more time trying to match the target notes with the violin than with the voice in the *Acquisition Aid* condition (M = 11.31, SD = 6 s more, see Figure 5A). On the contrary, the experts seemed to spend slightly more time with the voice than with the violin (M = 1.6, SD = 2.25 s more). Univariate tests of within-subject effects showed a significant effect of Instrument (violin > voice), *F*_(1)_ = 13.31, *p* = 0.001, η^2^ = 0.172, and an Instrument*Group interaction, *F*_(3)_ = 7.25, *p* < 0.0001, η^2^ = 0.254. Simple main effect analysis revealed a significant effect of Instrument in the univariate tests of within-subject effects for the experts (voice>violin), *F*_(1)_ = 18.25, *p* = 0.013, η^2^ = 0.36, and the ETG (violin > voice), *F*_(1)_ = 67.29, *p* < 0.0001, η^2^ = 0.77. No significant effect of Instrument was found for the FG or the CG.

Participants from the ETG tended to spend less time with the voice than participants from the FG (M = 9.4, SD = 9.29 s less). Those big differences were not seen in the violin condition where the ETG spent only 1.61 (SD = 9.13) seconds less than the FG. Independent samples *t*-test showed significant effects between the FG and ETG for the voice *t*_(36)_ = 2.93, *p* = 0.004. No significant differences were found for the violin.

Results for the number of requests at the *Acquisition Aid* condition showed how participants from the ETG tended, on average, to request more times the auditory feedback at the violin condition than at the voice condition (M = 0.92, SD = 0.51 times more, see Figure 5B). On the other hand, participants from the CG barely requested more feedback from the synthesizer either at the violin or at the voice condition. Univariate tests of within-subject effects showed a significant effect of Instrument (violin > voice), *F*_(1)_ = 26.67, *p* < 0.0001, η^2^ = 0.44, and an Instrument*Group interaction, *F*_(1)_ = 34.21, *p* < 0.0001, η^2^ = 0.50. Simple main effect analysis revealed a significant effect of Instrument in the univariate tests of within-subject effects for the ETG (violin>voice), *F*_(1)_ = 52.54, *p* < 0.0001, η^2^ = 0.71.

Finally, no significant correlations were found between the time spent or the number of attempts in *Acquisition Aid* with the error in cents at the *Transfer* condition. Neither for the violin nor for the voice.

## 4. Discussion

In this experiment, we have evaluated the use of different feedback modalities for learning intonation skills. We have compared real-time visual feedback and auditory feedback with a similar timbre for both violin and singing voice.

First of all, we needed to ensure that beginners were capable of controlling the pitch with the violin and, in any case, that their inability to do so was not an impediment to find and reproduce the demanded note in the fingerboard. Through the use of sound quality and bow kinematics descriptors validated in previous studies (Romaní et al., 2015; Giraldo et al., 2019; Blanco et al., 2021a), we investigated correlations between beginners' violin technique and intonation accuracy at the different conditions. No correlation was found. We also found that all groups of beginners improved their violin technique throughout the experiment in a similar way regardless to which group they belonged. Finally, we also found that beginners obtained lower absolute intonation errors in cents with the violin than with the voice. Even though beginners spent more time before the experiment learning violin technique than voice technique they learned the minimum necessary to control pitch production and realize sweeps through the fingerboard (which was the same technique required for their voice). That seems to reject the idea that differences in violin and singing voice technical skills may have influenced the intonation results.

Participants in this study produced an intonation error with the voice greater than the found in previous studies evaluating pitch-matching skills (Dalla Bella et al., 2007; Pfordresher and Brown, 2007; Wise and Sloboda, 2008; Pfordresher et al., 2010; Hutchins and Peretz, 2012; Berkowska and Dalla Bella, 2013). Hutchins and Peretz (2012), for example, reported an average singing absolute intonation error of 129 cents for non-musicians while in this study the absolute error was 217 cents. We believe that the choice of a pure tone synthesized sound used in this study may have caused a larger error, while the voice-like synthesizer sound used in the literature may reduce the intonation errors. The fact that the error with the violin was less than with the voice could be due to a possible greater timbral similarity of the violin with the synthesizer. Expert violinists also tended to be more accurate with the violin than with the voice, however, this could be due to increased experience with the instrument. On the other hand, Hutchins and Peretz (2012) found that the vocal tones tended to be matched with less accuracy than the tones produced with the slider, which could also have influenced the results of the experts. In Hutchins and Peretz (2012), musicians showed an average error of 2 cents with the slider and 17 cents with the voice while in this experiment experts showed an average error of 18 cents with the violin and 26.8 cents with the voice.

Although the accuracy with the violin was higher than with the voice, we did not find differences between the number of correct notes produced by each modality that oscillated between one and two correct notes (out of five) in the Baseline. Even assuming that the participants gave the same note in each attempt we could find similar results if that note was located between two of the target notes. To make sure that the participants were trying to hit the notes and not producing the same in each attempt, we calculated if there was some kind of correlation between the target notes and the produced notes. We found significant correlations for both the violin and the voice. Furthermore, we did not find significant differences in the absolute error of each note neither with the violin nor with the voice. This suggests that the frequency of the note was not a factor influencing the intonation accuracy. Interestingly, experts tended to show a trend to make flat errors both with the violin and the voice that was not seen in beginners. This contrasts with the results reported by Hutchins and Peretz (2012) where results with the slider showed no trend to flat or sharp error neither in musicians and non-musicians whereas results with the voice showed a trend to flat errors in both groups. Interestingly, Pfordresher and Brown (2007) did not report any prevailing tendency toward flat or sharp singing among poor-pitch singers. Although our participants might not be considered poor-pitch singers, the difficulty of the task (matching a pure-tone) may have led to similar behaviors. In relation to the experts, we hypothesize that pure tones could be perceived slightly flat compared with the timbre of the violin, although we cannot offer evidence of this fact.

Once the behavior of the participants in the Baseline condition had been studied, we proceeded to study their behavior in the rest of the blocks of the experiment and what possible influences the feedback received could exert on them.

Both FG and ETG improved significantly their results with both the violin and the voice when received visual or aural feedback at the *Acquisition post-Aid*. However, the FG was the only group that showed retention at both the *Acquisition pre-Aid* and the *Transfer* conditions at both modalities. As expected, neither the CG nor the experts improved among the session (in the case of the experts because their errors were minimal).

Despite the average error in cents at the *Acquisition post-Aid* for the voice was larger in the ETG than in the FG, the number of correct notes did not differ between groups (around four correct notes, which is also the average of correct notes of the experts). We can also see an improvement in the number of correct notes at the *Acquisition post-Aid* for the violin in the ETG compared with the rest of the conditions (around three correct notes out of five), although not as high as with the voice.

The fact that the CG participants were not able to match the notes, even after having the opportunity to try and listen to the synthesized pitch as many times as they wanted, confirms that the lack of trials was not the reason for the poor results beginners showed in their singing abilities. Both ETG and FG were able to match a similar number of notes when receiving help, either in the form of auditory feedback or in the form of visual feedback. However, only the group of participants who received visual feedback seemed to retain their results both at the *Acquisition pre-Aid* and the *Transfer* condition.

The FG spent more time at the *Acquisition Aid* trying to match the pitch than the rest of the groups in the voice condition. This seems to be confounded with the degree of improvement. However, no correlation between the duration or the number of attempts with the produced error at the *Transfer* condition was found neither for the violin nor the voice. A similar effect has been already reported in Hutchins and Peretz (2012). They found that participants tended to spend more time and make more attempts with the slider than with the voice. To determine whether the advantage of the slider condition compared with the voice condition was due to the number of attempts, they required participants to make a minimum number of voice responses comparable to those of the slider. No changes in their voice responses were found across their attempts. As Hutchins and Peretz (2012) suggested, the reason why participants tended to make few responses and spent less time with the voice is probably because participants determined that further responses would not aid their accuracy.

Welch (1984) and Hutchins and Peretz (2012) showed how online visual feedback of the pitch does not improve by itself the pitch-matching results of participants. It seems that there is the need for objective information on the screen regarding how far or close is the produced pitch from the correct result, just as in linear positioning tasks, where subjects had to move an object toward a target out of sight with no time limit. However, our participants were not “tone deaf” and some of the hypothesized reasons why they may not be able to pitch-match our synthesized tone were because of their inability to translate from one timbre to another (Hutchins and Peretz, 2012). By offering them aural feedback of a similar timbre to the one produced, we expected to help them to establish the parameters of the translation by themselves, without the need for an unmistakable sign of “correct” or “incorrect.” The fact that on average, participants from the ETG were able to produce a higher number of correct notes at the *Acquisition post-Aid*, highlights that their difficulties were in part based on a pitch-translation problem. Nonetheless, this technique did not seem to help participants to retain the new mapping.

As we mentioned before, timbral similarity had stronger effects for the voice than for the violin. It is possible that participants were more able to be in tune with a human voice due to implicit imitation skills (Buccino et al., 2004; Christiner and Reiterer, 2013). The fact that participants in the ETG were capable of finding the correct pitch in less time than the FG during the *Acquisition Aid* and requested feedback a lesser number of times suggests this was the case. For some participants of the ETG, finding the pitch after hearing the vocal sounds was an almost automatic task done without effort while, for the FG, they had to explore sweeping their voice through the screen until the objective pitch was matched.

Humans are capable to imitate arbitrary sounds thanks to similar mechanisms that operate in other animal species through auditory-guided vocal learning/imitation (Brown et al., 2004; Fitch, 2006). It is known that both auditory memories and aural feedback interact to guide vocal imitation which probably explains why it was easier for participants to imitate human voices than violin sounds. Auditory-guided imitation thus, although helped participants to improve in their task, did not seem to help participants to establish and retain the new mapping between timbers. It is possible that both the objective visual measures and the experience of exploring the pitch space with their voice in an explicit manner, helped participants to understand how they got where they wanted to go, strengthening the schema and favoring retention. Another possibility is that although participants from the ETG were able to perform the task correctly in the *Acquisition post-Aid*, the level of confidence of their chosen answers could have been less as those of the FG, impairing them to learn the mapping between one timbre and the other. Future research could address this issue by offering binary feedback (right/wrong) to some participants in the equal-timbre group after their chosen answers. For now, as already pointed out by Welch (1984), reward seems almost indispensable when learning to translate from one timbre to another.

## 5. Conclusions

In summary, we can list some of the main findings of this study:

1. We found that auditory feedback in the form of timbre-similarity helped more to sing in tune with the voice rather than with the violin. Participants from the ETG also spent less time choosing their answers than participants from the FG at the *Acquisition pre-Aid*. We suggest that implicit imitation skills, above timbre-similarity, may also play an important role in matching the desired pitch.
2. Participants from the ETG were not able to retain their results for the rest of the conditions where auditory feedback was removed.
3. We have revalidated the importance of real-time visual feedback and KR for learning intonation. Participants from the FG were the only ones which improved their results significantly at the *Transfer* condition both with the violin and the voice.

## Data Availability Statement

The datasets generated for this study can be found in https://doi.org/10.5281/zenodo.4630144 and http://doi.org/10.5281/zenodo.4553940.

## Ethics Statement

The studies involving human participants were reviewed and approved by the Conservatoires UK Research Ethics committee on 04/04/2017, following the guidelines of the British Psychological Society. The patients/participants provided their written informed consent to participate in this study.

## Author Contributions

AB and RR designed the methodology of the study. AB recorded, processed, and analyzed the audio data, and wrote the paper. RR supervised the study and contributed to the writing of the paper. ST supervised the statistical methods used and contributed to the writing of the paper. All authors contributed to the article and approved the submitted version.

## Conflict of Interest

The authors declare that the research was conducted in the absence of any commercial or financial relationships that could be construed as a potential conflict of interest.

## Footnotes

^1^telmi.upf.edu

^2^https://youtu.be/mUz8fIc1FaY
